# ﻿Four new species of the genus *Camptoscaphiella* Caporiacco, 1934 (Araneae, Oonopidae) from Xizang, China

**DOI:** 10.3897/zookeys.1208.127725

**Published:** 2024-07-26

**Authors:** Xiaohan Wang, Yanfeng Tong, Shuqiang Li

**Affiliations:** 1 College of Life Science, Shenyang Normal University, Shenyang 110034, Liaoning, China Shenyang Normal University Shenyang China; 2 Institute of Zoology, Chinese Academy of Sciences, Beijing 100101, China Institute of Zoology, Chinese Academy of Sciences Beijing China

**Keywords:** Aranei, biodiversity, goblin spiders, Himalaya, spider, taxonomy, Tibet

## Abstract

Four new species of the genus *Camptoscaphiella* Caporiacco, 1934 are described from Xizang, China, i.e., *C.metok* Tong & Li, **sp. nov.** (♂), *C.shannan* Tong & Li, **sp. nov.** (♂♀), *C.trifoliata* Tong & Li, **sp. nov.** (♂♀) and *C.zayu* Tong & Li, **sp. nov.** (♂♀). Morphological descriptions, photographic illustrations and a distribution map of the four new species are given.

## ﻿Introduction

The family Oonopidae is one of the most diverse spider families worldwide, comprising 115 extant genera and 1952 species, and 3 fossil genera and 45 species (WSC 2024). *Camptoscaphiella* Caporiacco, 1934 is a small genus, distributed mainly in montane tropical and subtropical regions in Asia, mostly within the Himalayan Plateau (WSC 2024). Baehr and Ubick (2010) revised this genus, with two species re-described and nine new species described from China, India, Nepal, Sri Lanka and Thailand, and a key to the known 15 species provided. Two other species were later recorded in the Pacific Island of New Caledonia (Baehr and Harvey 2013) and five species were documented from Yunnan, China (Huang et al. 2021; Wang et al. 2023). Altogether, 23 species have been recorded in the world (WSC 2024).

Up to now, seven species belonging to three genera of oonopid spiders have been recorded in Xizang of China (Cheng et al. 2021; Tong et al. 2023). Although there are no records of the genus *Camptoscaphiella* from Xizang, many species have been described from the adjacent areas, including one from Bhutan, three from India, seven from Nepal, and eight from Yunnan Province of China (Tong and Li 2007; Baehr and Ubick 2010; Huang et al. 2021; Wang et al. 2023).

In this paper, *Camptoscaphiella* is recorded for the first time from Xizang and four new species of the genus are described and photographed.

## ﻿Materials and methods

The specimens were examined using a Leica M205C stereomicroscope. Details were studied under an Olympus BX51 compound microscope. Photos were captured with a Canon EOS 550D zoom digital camera (18 megapixels) mounted on an Olympus BX51 compound microscope. Endogynes were cleared in lactic acid. Scanning electron microscope (SEM) images were taken under high vacuum with a Hitachi S-4800 after critical-point drying and gold-palladium coating. All measurements were taken using an Olympus BX51 compound microscope and are in millimeters. The type material is deposited at the Shenyang Normal University (SYNU) in Shenyang, China.

The following abbreviations are used in the text and figures: **ALE** = anterior lateral eyes; **ap** = apodemes; **as** = anterior sclerite; **cd** = copulatory duct; **dp** = dorsal process; **mp** = median plate; **mpr** = median process; **np** = narrow process; **PLE** = posterior lateral eyes; **PME** = posterior median eyes; **pro** = prolateral outgrowth; **rlf** = retrolateral fold; **tfp** = trifurcate process; **tls** = thread-like structure; **vo** = ventral outgrowth; **vp** = ventral process; **wlo** = wing-like outgrowth.

## ﻿Taxonomy

**Family Oonopidae Simon, 1890**

### ﻿Genus Camptoscaphiella

Taxon classificationAnimaliaAraneaeOonopidae

Caporiacco, 1934

2ED1307A-BD55-5620-BA97-EE67EFFA5F76

#### Type species.

*Camptoscaphiellafulva* Caporiacco, 1934, by monotypy.

#### Diagnosis.

Males of this genus are similar to those of *Opopaea* Simon, 1892 in the extremely large, club-shaped palpal patella, but can be distinguished by the spination of the legs I–II (tibia I and II with four pairs of long spines, and metatarsus I and II with two pairs of long spines) vs. without spination; the cymbium not fused with the bulb vs. fused; and the reduced abdominal scuta vs. complete scuta. Females of this genus are similar to those of *Ischnothyreus* Simon, 1893, but can be separated by lacking the distinct, darkly sclerotized, strongly winding duct of endogyne and the uniquely shaped atrium.

#### Distribution.

China (Xizang, Yunnan), South Asia (Bhutan, India, Nepal, Pakistan, Sri Lanka), Southeast Asia (Thailand) and New Caledonia.

### ﻿ Camptoscaphiella metok

Taxon classificationAnimaliaAraneaeOonopidae

Tong & Li sp. nov.

46B98DF7-F98D-58F5-8B0C-C9636C9B0323

https://zoobank.org/4B0D244B-5E71-48DD-9D92-434C7A0B2AD1

Figs 1 , 2 , 12

#### Material examined.

***Holotype*** China • ♂ (SYNU-1134); Xizang, Nyingchi, nr. Metok Co.; 29°19.382'N, 95°19.016'E, 980 m; 2.VIII.2013; Y. Lin leg.

***Paratype***: China • ♂ (SYNU-1135); same data as for holotype, Motuo Petrol Station; 22.IX.2013; Z. Gao leg.

#### Diagnosis.

**Figure 1.**
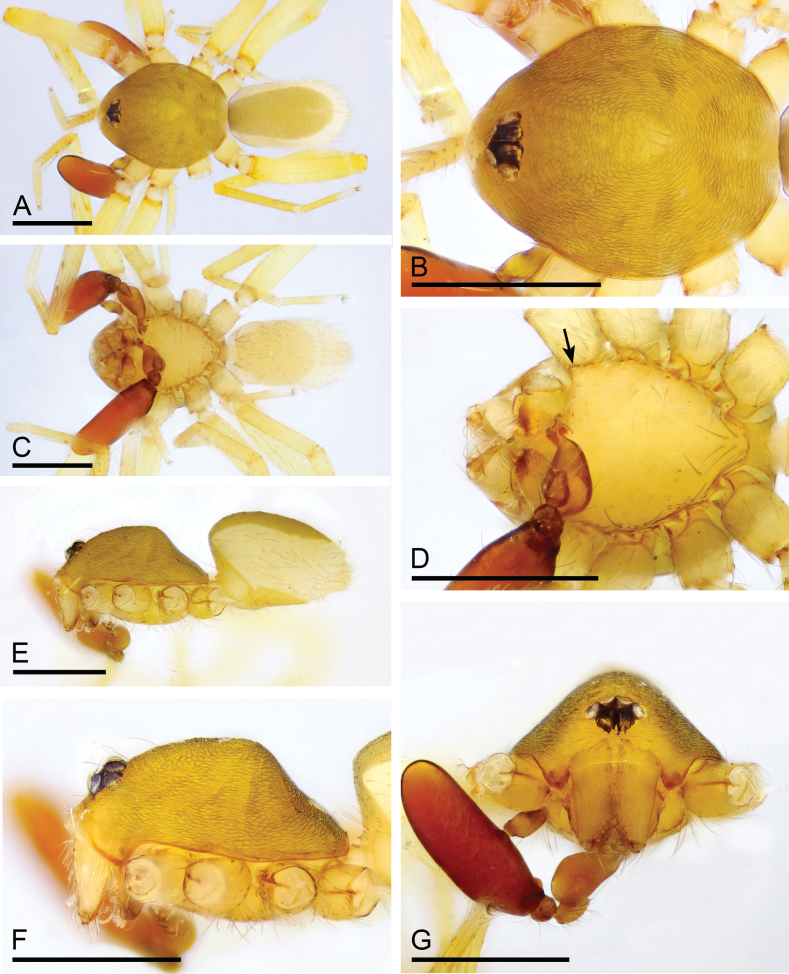
*Camptoscaphiellametok* sp. nov., male holotype **A, C, E** habitus, dorsal, ventral and lateral views **B, D, F, G** prosoma, dorsal, ventral, lateral and anterior views, arrow shows the pointed anterolateral bumps. Scale bars: 0.4 mm (**A–G**).

**Figure 2.**
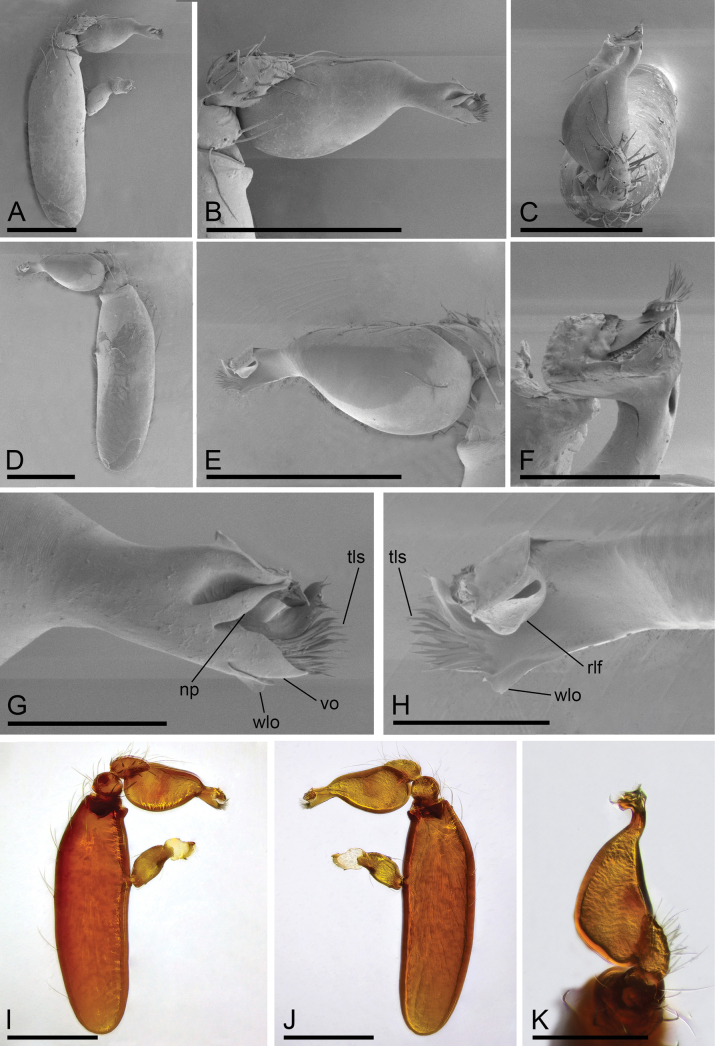
*Camptoscaphiellametok* sp. nov., male left palp **A, I** prolateral view **B, E** bulb, prolateral and retrolateral views **C, K** dorsal view **D, J** retrolateral view **F, G, H** distal part of bulb, dorsal, prolateral and retrolateral views. Abbreviations: np = narrow process; rlf = retrolateral fold; tls = thread-like structure; vo = ventral outgrowth; wlo = wing-like outgrowth. Scale bars: 0.2 mm (**A–E, I–K**); 0.05 mm (**F–H**).

This new species is similar to *C.linyejiei* Tong & Li, 2021, but can be distinguished by their normal-sized eyes (Fig. 1B, G) vs. reduced (Huang et al. 2021: fig. 4B, G); and the thread-like structure (tls) on the tip of the psemblous (Fig. 2G, H), vs. lacking; and having a plate-like process (Huang et al. 2021: fig. 5G, H).

#### Description.

**Male** (holotype). ***Body***: uniformly colored, yellow; habitus as in Fig. 1A, C, E; length 1.29. ***Carapace*** (Fig. 1B, F): 0.66 long, 0.54 wide; pars thoracica strongly elevated in lateral view, entire surface finely reticulated. ***Eyes*** (Fig. 1B, G): ALE: 0.05; PME: 0.04; PLE: 0.04; ALE circular, PME oval, PLE oval; posterior eye row procurved from both above and front; ALE separated by less than one radius. ***Clypeus*** (Fig. 1F, G): margin unmodified, straight in front view, sloping forward in lateral view. ***Mouthparts*** (Fig. 1D, G): chelicerae unmodified; anterior-median part of endites strongly sclerotized. ***Sternum*** (Fig. 1D): as long as wide, with pointed anterolateral bumps. ***Abdomen*** (Fig. 1A, C, E): 0.63 long, 0.34 wide; oval, scuta pale orange; dorsal scutum covering about 5/6 of abdomen length, about 3/4 of abdomen width, fused to epigastric scutum; postgastric scutum covering about 3/4 of abdominal venter. ***Palp*** (Fig. 2A–K): reddish brown; patella extremely long club-shaped, length/width = 3.04, about 5.6 times longer than femur, and 2.5 times longer than bulb; cymbium narrow (length/width = 1.68) in dorsal view; distal part of bulb with narrow process (np), broad ventral outgrowth (vo), a narrow wing-like outgrowth (wlo), a small retrolateral fold (rlf) and cluster of thread-like structure (tls).

**Female.** Unknown.

#### Notes.

Seven species [*C.glenniei* (Fage, 1946) from India, *C.martensi* Baehr, 2010, *C.panchthar* Baehr, 2010, *C.silens* Brignoli, 1976, *C.strepens* Brignoli, 1976 and *C.taplejung* Baehr, 2010 from Nepal and *C.gunsa* Baehr, 2010 from India and Nepal] in adjacent areas are known only from females and this new species can be potentially conspecific with one of them.

#### Etymology.

The specific name refers to the type locality and is a noun in apposition.

#### Distribution.

Known only from the type locality.

### ﻿ Camptoscaphiella shannan

Taxon classificationAnimaliaAraneaeOonopidae

Tong & Li sp. nov.

A8533E44-22F6-5087-9437-562854708E23

https://zoobank.org/6EC89B40-CFA0-4E7C-8F75-9102C4BF40F3

Figs 3 , 4 , 5 , 12

#### Material examined.

***Holotype*** China • ♂ (SYNU-1143); Xizang, Shannan City, Cona Co., Lemenba Ethnic Township, 17–20 km section from Lewang Bridge to Liulian Hwy; 27°47.700'N, 91°46.417'E, 3706 m; 5.VI.2016; J. Wu leg.

***Paratypes***: China • ♀ (SYNU-1144); same data as for holotype • 6♀ (SYNU-1123–1128); same data as for holotype.

#### Diagnosis.

**Figure 3.**
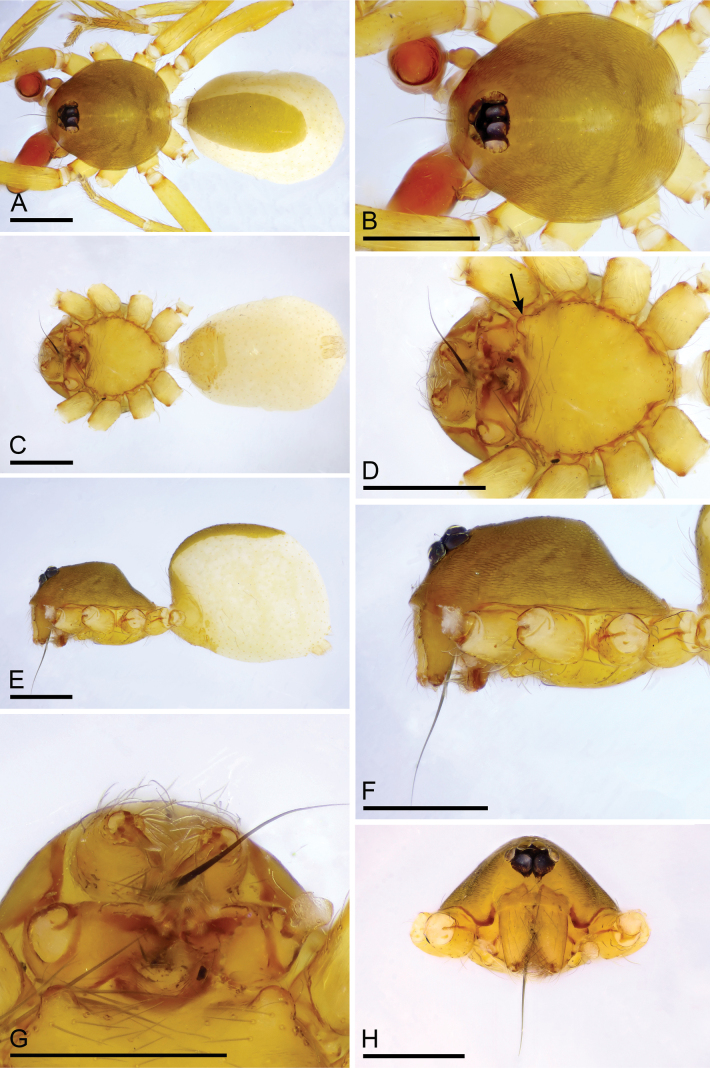
*Camptoscaphiellashannan* sp. nov., male holotype **A, C, E** habitus, dorsal, ventral and lateral views **B, D, F, H** prosoma, dorsal, ventral, lateral and anterior views, arrow shows the pointed anterolateral bumps **G** labium and endites, ventral view. Scale bars: 0.4 mm (**A–G**).

**Figure 4.**
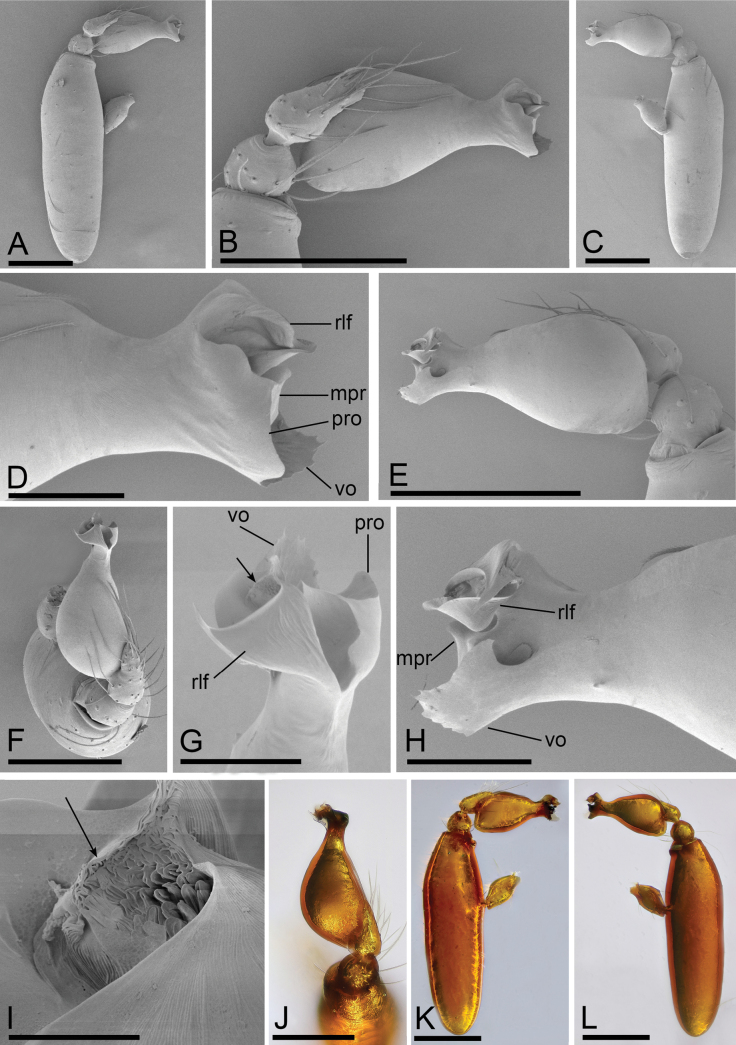
*Camptoscaphiellashannan* sp. nov., male left palp **A, K** prolateral view **B, E** bulb, prolateral and retrolateral views **C, L** retrolateral view **D, G, H** distal part of bulb, prolateral, dorsal and retrolateral views **F, J** dorsal view **I** same as**G** showing the details (arrow). Abbreviations: mpr = median process; pro = prolateral outgrowth; rlf = retrolateral fold; vo = ventral outgrowth. Scale bars: 0.2 mm (**A–C, E, F, J–L**); 0.05 mm (**D, G, H**); 0.01 mm (**I**).

**Figure 5.**
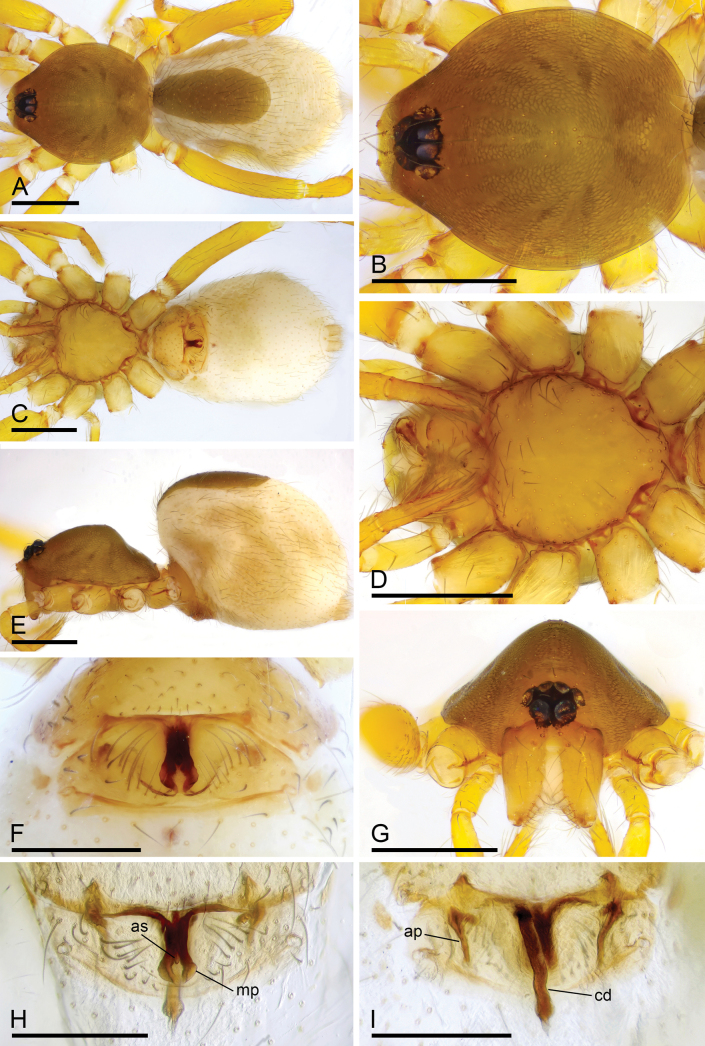
*Camptoscaphiellashannan* sp. nov., female paratype (SYNU-1144) **A, C, E** habitus, dorsal, ventral and lateral views **B, D, G** prosoma, dorsal, ventral and anterior views **F** epigastric region, ventral view **H, I** endogyne, ventral and dorsal views. Abbreviations: ap = apodemes; as = anterior sclerite; cd = copulatory duct; mp = median plate. Scale bars: 0.4 mm (**A–E, G**); 0.2 mm (**F, H, I**).

The new species is similar to *C.tuberans* Tong & Li, 2007 and can be distinguished by the single strong bristle behind cheliceral paturons (Fig. 3D–H), vs. bristle absent (Tong and Li 2007: fig. 23); the strongly sclerotized median process (mpr) and a broad ventral outgrowth (vo) of the psembolus (Fig. 4D, H, K) vs. lacking the median process and having instead a very narrow, bifurcated ventral outgrowth (Tong and Li 2007: figs 25–27); and narrow forcipate median plate (mp) of endogyne (Fig. 5F, H) vs. circular median plate (Tong and Li 2007: fig. 22).

#### Description.

**Male** (holotype). ***Body***: uniformly colored, yellow; habitus as in Fig. 3A, C, E; length 1.83. ***Carapace*** (Fig. 3B, F): 0.81 long, 0.72 wide; pars thoracica slightly elevated in lateral view, entire surface finely reticulated. ***Eyes*** (Fig. 3B, H): ALE: 0.08; PME: 0.06; PLE: 0.06; ALE circular, PME oval, PLE oval; posterior eye row straight from above, procurved from front; ALE separated by less than one radius. ***Clypeus*** (Fig. 3F, H): margin unmodified, straight in front view, sloping forward in lateral view. ***Mouthparts*** (Fig. 3G, H): with single strong bristle behind paturons; anterior-median part of the endites strongly sclerotized. ***Sternum*** (Fig. 3D): as long as wide, with pointed anterolateral bumps. ***Abdomen*** (Fig. 3A, C, E): 1.02 long, 0.73 wide; oval, scuta pale orange; dorsal scutum covering about 2/3 of abdomen, 1/2 of abdomen width, fused to epigastric scutum; postgastric scutum small. ***Palp*** (Fig. 4A–L): reddish brown; patella extremely long club-shaped, length/width = 3.30, about 5.8 times longer than femur, and 3.2 times longer than bulb, cymbium narrow (length/width = 1.90) in dorsal view; psembolus with prolateral and ventral outgrowths (pro and vo respectively), strongly sclerotized median process (mpr) and small retrolateral fold (rlf).

**Female** (SYNU-1144). As in male except for the following. ***Body***: habitus as in Fig. 5A, C, E; length 1.98. ***Carapace***: 0.86 long, 0.73 wide. ***Eyes***: ALE 0.07; PME 0.05; PLE 0.07. ***Abdomen***: 1.12 long, 0.80 wide. Postgaster (Fig. 5F, H): with stick-like anterior sclerite (as) and forcipate median plate (mp). ***Endogyne*** (Fig. 5I): copulatory duct (cd) long, narrow, straight with tip well beyond postgastric scutum; apodemes (ap) short.

#### Etymology.

The specific name refers to the type locality and is a noun in apposition.

#### Distribution.

Known only from the type locality.

### ﻿ Camptoscaphiella trifoliata

Taxon classificationAnimaliaAraneaeOonopidae

Tong & Li sp. nov.

1F5069E6-4592-54BC-90BC-2C8E807459A3

https://zoobank.org/C007206E-B7E4-46C9-A4F9-59DEE08A0442

Figs 6 , 7 , 8 , 12

#### Material examined.

***Holotype*** China • ♂ (SYNU-1145); Xizang, Rikaze City, Jilong Co., Zalong Vill.; 28°22.865'N, 85°21.158'E, 2715 m; 31.VII.2014; Y. Li leg.

***Paratypes***: China • 1 ♀ (SYNU-1100); Rikaze City, Dingjie Co., Chentang Town; 27°54.875'N, 87°28.869'E, 3267 m; 3.VIII.2014; Y. Li leg. • 1 ♂ (SYNU-1099); Rikaze City, Dingri Co., Rongxia Town; 28°03.450'N, 86°21.148'E, 3383 m; 27.VII.2014; Y. Li leg. • 2 ♂ (SYNU-1054–1055); Nyingchi, Bayi Distr., Bayi Town, Biri Mt; 28°51.334'N, 94°47.941'E, 2900 m; 11.VII.2013; Y. Lin leg. • 11♀ (SYNU-1057–1067); same data as above • 4♀ (SYNU-1146–1149); same data as above • 2 ♂ 4 ♀ (SYNU-1113–1118); Nyingchi, Bayi Distr., Lulang Town; 29°41.449'N, 94°43.605'E, 3530 m; 14.VII.2013; Y. Lin leg. • 9 ♂ 2 ♀ (SYNU-1101–1112); Nyingchi, Bayi Distr., Lulang Town; 29°21.449'N, 94°43.605'E, 3530 m; 14.VII.2013; Q. Cao leg. • 5 ♂ 1 ♀ (SYNU-1068–1073); Nyingchi, Mainling Co.; 29°13.310'N, 94°13.309'E, 3050 m; 13.VIII.2013; Y. Lin leg. • 2 ♂ 1 ♀ (SYNU-1152–1154); same data as above • 6 ♂ 4 ♀ (SYNU- 1074–1083); Nyingchi, Mainling Co.; 29°12.316'N, 94°12.649'E, 3060 m; 13.VIII.2013; Y. Lin leg. • 4♂10♀ (SYNU-1084–1097); Nyingchi, Mainling Co., Pai Town; 29°30.264'N, 94°53.868'E, 3321 m; 6.VIII.2015; J. Wu leg.

#### Diagnosis.

**Figure 6.**
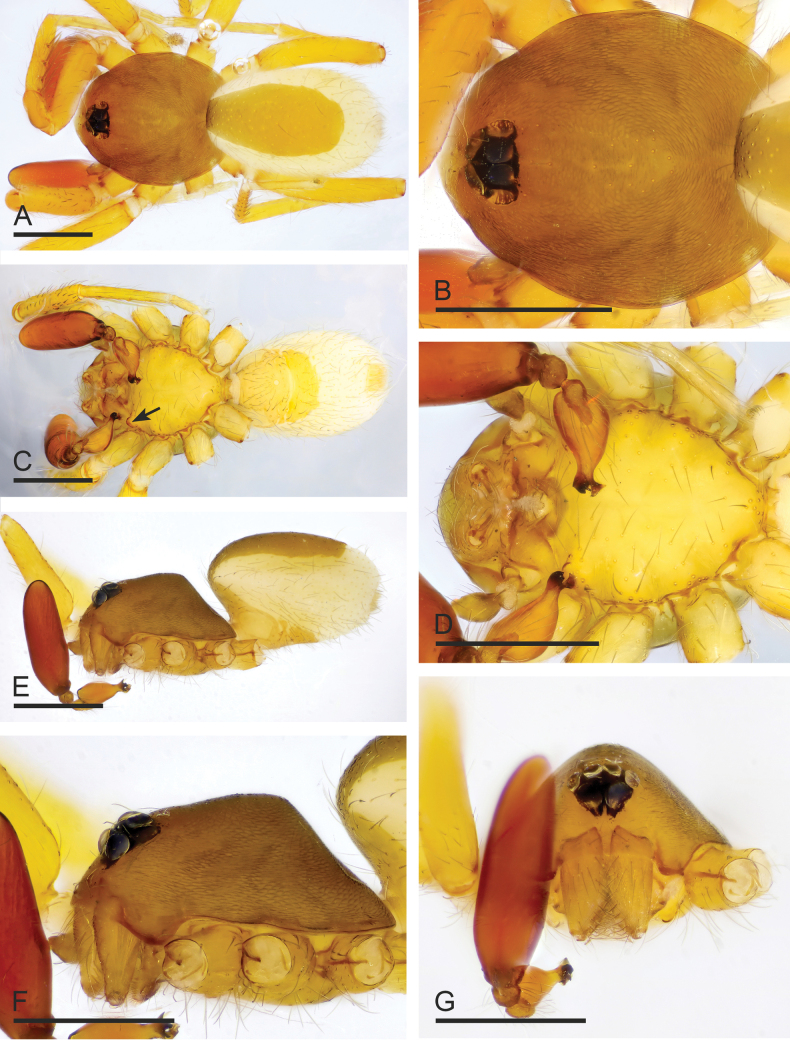
*Camptoscaphiellatrifoliata* sp. nov., male holotype **A, C, E** habitus, dorsal, ventral and lateral views, arrow shows the pointed anterolateral bumps **B, D, F, G** prosoma, dorsal, ventral, lateral and anterior views. Scale bars: 0.4 mm (**A–G**).

**Figure 7.**
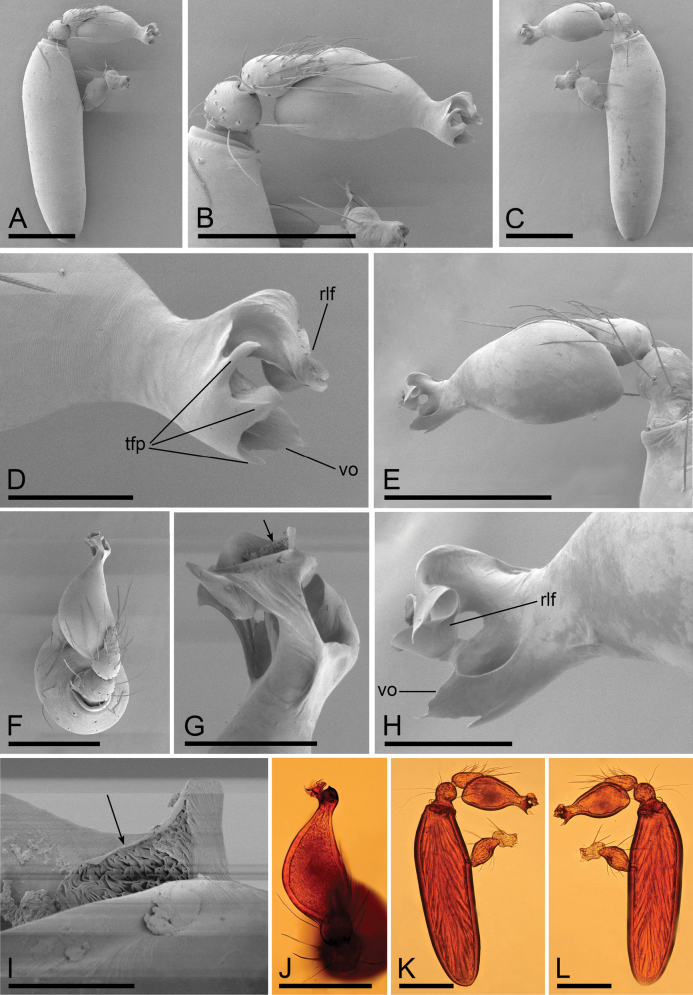
*Camptoscaphiellatrifoliata* sp. nov., male left palp **A, K** prolateral view **B, E** bulb, prolateral and retrolateral views **C, L** retrolateral view **D, G, H** distal part of bulb, prolateral, dorsal and retrolateral views **F, J** dorsal view **I** same as**G** showing the details (arrow). Abbreviations: rlf = retrolateral fold; tfp = trifurcate process; vo = ventral outgrowth. Scale bars: 0.2 mm (**A–C, E, F, J–L**); 0.05 mm (**D, H, G**); 0.01 mm (**I**).

**Figure 8.**
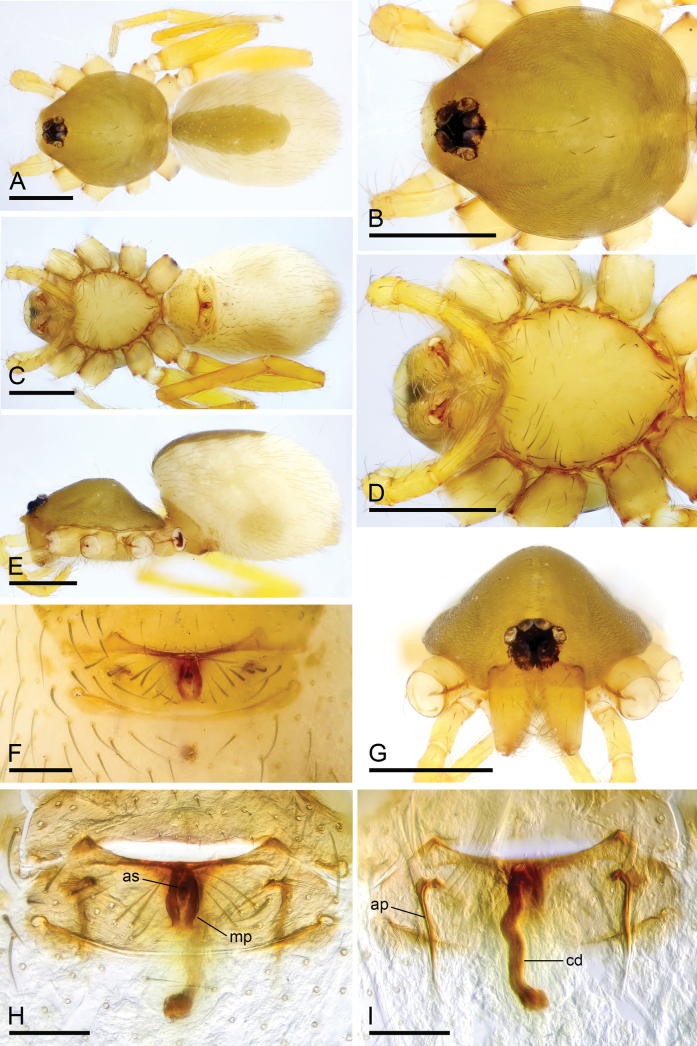
*Camptoscaphiellatrifoliata* sp. nov., female paratype (SYNU-1146) **A, C, E** habitus, dorsal, ventral and lateral views **B, D, G** prosoma, dorsal, ventral and anterior views **F** epigastric region, ventral view **H, I** endogyne, ventral and dorsal views. Abbreviations: ap = apodemes; as = anterior sclerite; cd = copulatory duct; mp = median plate. Scale bars: 0.4 mm (**A–E, G**); 0.2 mm (**F, H, I**).

Males of this new species are similar to those of *C.yujufeng* Tong & Yang, 2023, but can be distinguished by lacking a cluster of black, strong setae on the labium (Fig. 6D) vs. setae cluster present (Wang et al. 2023: fig. 5D) and psemblous with trifurcate process (tfp, Fig. 7D, K) vs. with semicircular, prolateral rim and trifurcate ventral process (Wang et al. 2023: fig. 6E, J). Females of the new species are similar to those of *C.zayu* sp. nov., but can be distinguished by the fusiform median plate (Fig. 8H) vs. pear-shaped (Fig. 11H) and the long copulatory duct, which extends beyond the postgastric scutum (Fig. 8I) vs. just reaching groove between posterior spiracles (Fig. 11I).

#### Description.

**Male** (holotype). ***Body***: uniformly colored, yellowish brown; habitus as in Fig. 6A, C, E; length 1.75. ***Carapace*** (Fig. 6B, F): 0.84 long, 0.68 wide; pars thoracica strongly elevated in lateral view, entire surface finely reticulated. ***Eyes*** (Fig. 6B, G): ALE: 0.07; PME: 0.05; PLE: 0.04; ALE circular, PME oval, PLE oval; posterior eye row straight from above, procurved from front; ALE separated by less than one radius. ***Clypeus*** (Fig. 6F, G): margin unmodified, straight in front view, sloping forward in lateral view. ***Mouthparts*** (Fig. 6D, G): chelicerae unmodified; anterior-median part of the endites slightly sclerotized. ***Sternum*** (Fig. 6D): as long as wide, with pointed anterolateral bumps. ***Abdomen*** (Fig. 6A, C, E): 0.91 long, 0.53 wide; oval, scuta pale orange; dorsal scutum covering about 3/4 of abdomen length, about 2/3 of abdomen width, fused to epigastric scutum; postgastric scutum small, covering about 1/2 of abdominal venter. ***Palp*** (Fig. 7A–L): reddish brown; patella extremely long club-shaped, length/width = 3.41, about 5.5 times longer than femur, and 2.5 times longer than bulb; cymbium (length/width = 1.92) narrow in dorsal view; psembolus with trifurcate process (tfp), ventral outgrowth (vo) and small retrolateral fold (rlf).

**Female** (SYNU-1100). As in male except for the following. ***Body***: habitus as in Fig. 8A, C, E; length 1.94. ***Carapace***: 0.83 long, 0.74 wide. ***Eyes***: ALE 0.07; PME 0.05; PLE 0.06. ***Abdomen***: 1.11 long, 0.76 wide. Postgaster (Fig. 8F, H): with rounded anterior sclerite (as) and fusiform median plate (mp). ***Endogyne*** (Fig. 8I): copulatory duct (cd) long, narrow, sinuous with tip well beyond postepigastric scutum; apodemes (ap) slender.

#### Etymology.

The specific name, derived from Latin word *trifoliatus*, refers to the three-forked processes on the distal part of the bulb; adjective.

#### Distribution.

Known only from the type locality.

### ﻿ Camptoscaphiella zayu

Taxon classificationAnimaliaAraneaeOonopidae

Tong & Li sp. nov.

7E5B7875-7328-50B3-8BDE-9F613C0398E2

https://zoobank.org/FC362C41-6FA7-450F-856B-4E16980715DD

**Figure 9.**
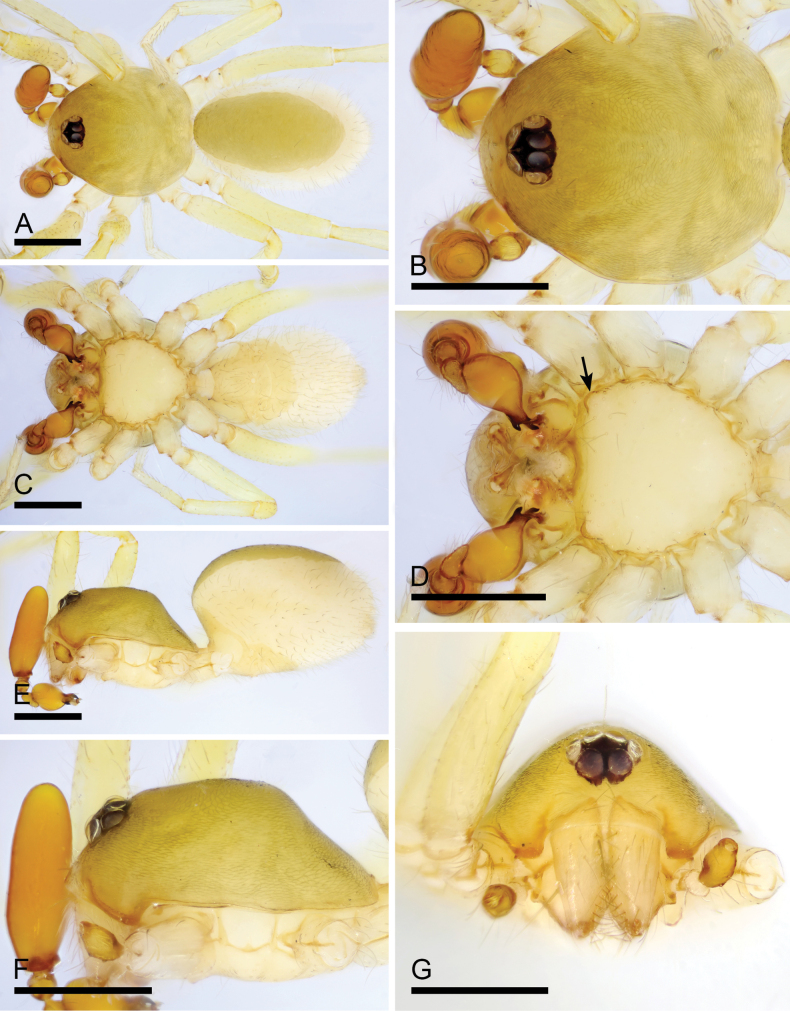
*Camptoscaphiellazayu* sp. nov., male holotype **A, C, E** habitus, dorsal, ventral and lateral views **B, D, F, G** prosoma, dorsal, ventral, lateral and anterior views, arrow shows the pointed anterolateral bumps. Scale bars: 0.4 mm (**A–G**).

**Figure 10.**
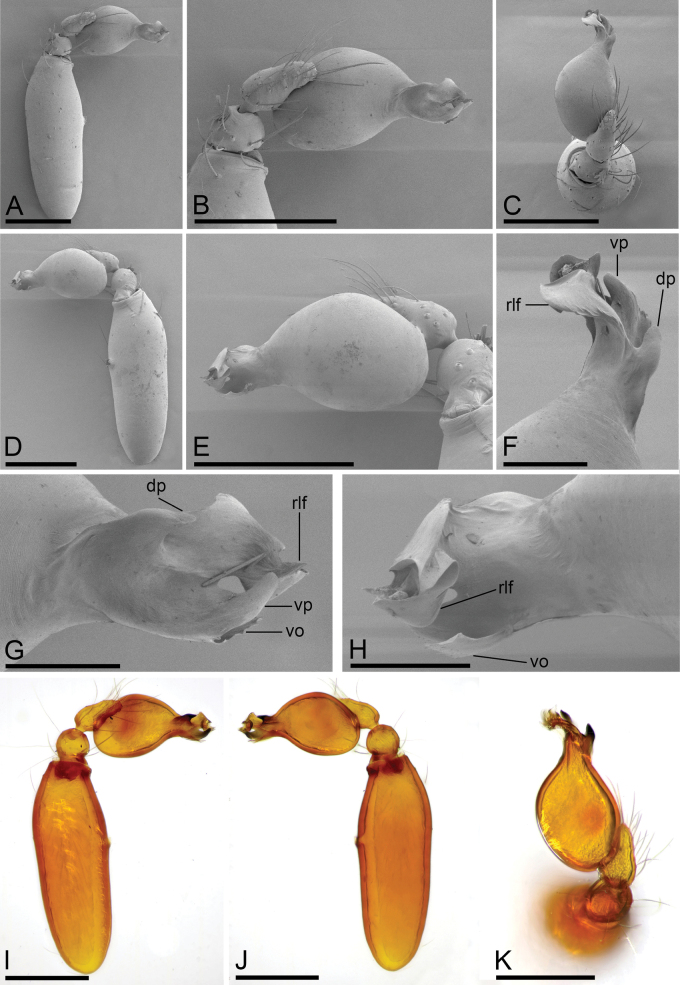
*Camptoscaphiellazayu* sp. nov., male left palp **A, I** prolateral view **B, E** bulb, prolateral and retrolateral views **C, K** dorsal view **D, J** retrolateral view **F, G, H** distal part of bulb, dorsal, prolateral and retrolateral views. Abbreviations: dp = dorsal process; rlf = retrolateral fold; vo = ventral outgrowth; vp = ventral process. Scale bars: 0.2 mm (**A–E, I–K**); 0.05 mm (**F–H**).

**Figure 11.**
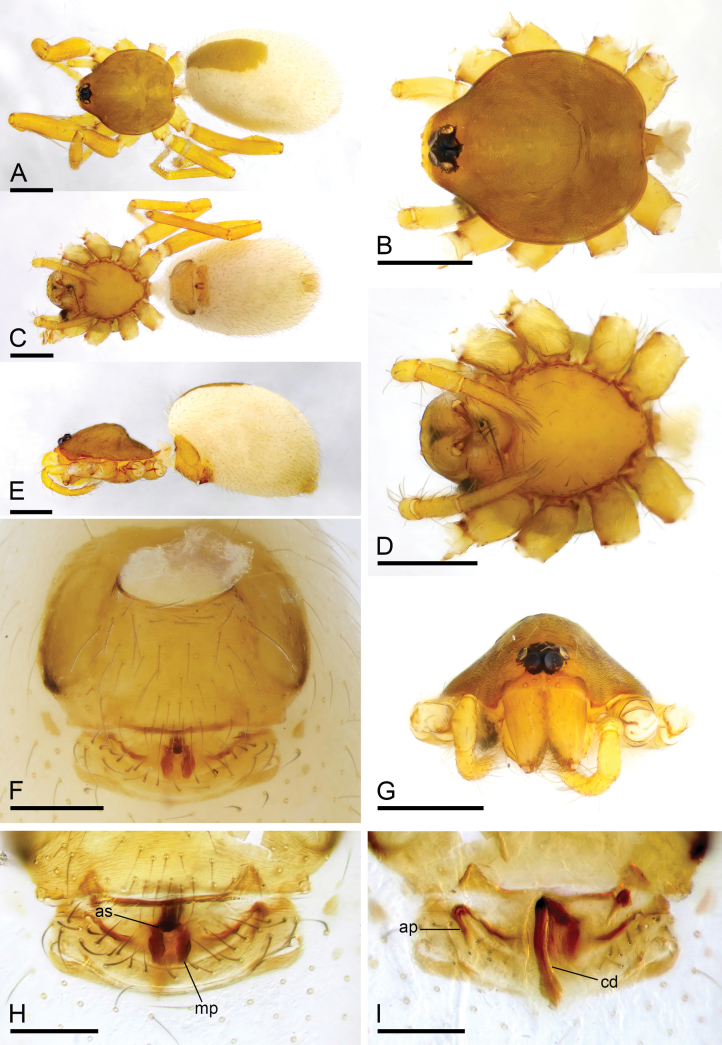
*Camptoscaphiellazayu* sp. nov., female paratype (SYNU-1142) **A, C, E** habitus, dorsal, ventral and lateral views **B, D, G** prosoma, dorsal, ventral and anterior views **F** epigastric region, ventral view **H, I** endogyne, ventral and dorsal views. Abbreviations: ap = apodemes; as = anterior sclerite; cd = copulatory duct; mp = median plate. Scale bars: 0.4 mm (**A–E, G**); 0.2 mm (**F, H, I**).

**Figure 12.**
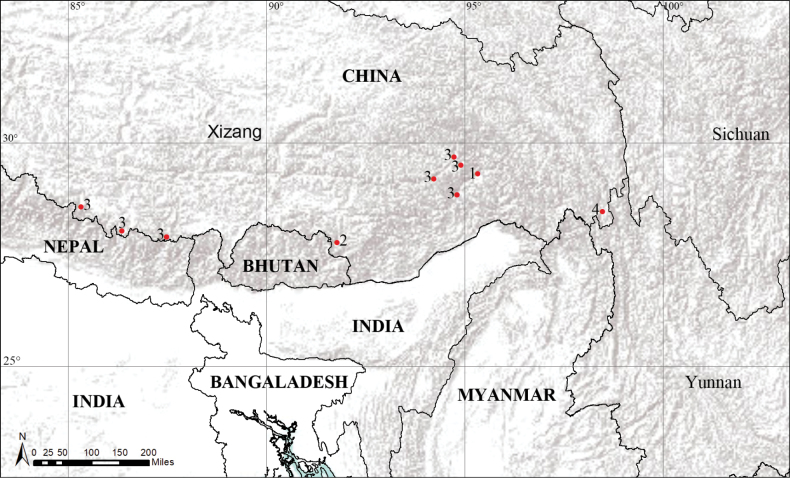
Distribution records of four new species of the genus *Camptoscaphiella* from Xizang, China. 1. *C.metok* sp. nov. 2. *C.shannan* sp. nov. 3. *C.trifoliata* sp. nov. 4. *C.zayu* sp. nov.

Figs 9 , 10 , 11 , 12

#### Material examined.

***Holotype*** China • ♂ (SYNU-1141); Xizang, Zayu Co., Chawalong Town, Long Vill.; 28°28.941'N, 98°28.193'E, 2883 m; 8.IX.2014; J. Liu leg.

***Paratypes*.** China • 1 ♀ (SYNU-1142); same data as for holotype.

#### Diagnosis.

The new species is similar to the type species, *C.fulva* Caporiacco, 1934, but can be distinguished by the strongly sclerotized dorsal (dp) and ventral (vp) processes of the psembolus (Fig. 10G, I) vs. with two spinelike processes (Baehr and Ubick 2010: figs 161–163), and long copulatory duct (cd, Fig. 11H, I) vs. short inverted droplet-shaped copulatory duct (Baehr and Ubick 2010: figs 182, 183).

#### Description.

**Male** (holotype). ***Body***: uniformly colored, pale yellow; habitus as in Fig. 9A, C, E; length 1.91. ***Carapace*** (Fig. 9B, F): 0.85 long, 0.78 wide; pars thoracica strongly elevated in lateral view, whole surface finely reticulated. ***Eyes*** (Fig. 9B, G): ALE: 0.08; PME: 0.06; PLE: 0.06; ALE circular, PME oval, PLE oval; posterior eye row procurved from both above and front; ALE separated by less than one radius. ***Clypeus*** (Fig. 9F, G): margin unmodified, straight in front view, sloping forward in lateral view. ***Mouthparts*** (Fig. 9D, G): chelicerae unmodified; anterior-median part of the endites slightly sclerotized. ***Sternum*** (Fig. 9D): as long as wide, with pointed anterolateral bumps. ***Abdomen*** (Fig. 9A, C, E): 1.06 long, 0.68 wide; oval, scuta pale orange; dorsal scutum covering about 5/6 of abdomen length, about 3/4 of abdomen width, fused to epigastric scutum; postgastric scutum small, covering about 1/2 of abdominal venter. ***Palp*** (Fig. 10A–K): reddish brown; patella extremely long club-shaped, length/width = 2.96, about 4.2 times longer than femur, and 2.5 times longer than bulb; cymbium narrow (length/width = 1.64) in dorsal view; psembolus with strongly sclerotized dorsal process (dp), strongly sclerotized ventral process (vp), broad ventral outgrowth (vo) and a small retrolateral fold (rlf).

**Female** (SYNU-1142). As in male except for the following. ***Body***: habitus as in Fig. 11A, C, E; length 2.37. ***Carapace***: 0.92 long, 0.81 wide. ***Eyes***: ALE 0.07; PME 0.05; PLE 0.05. ***Abdomen***: 1.45 long, 0.90 wide. ***Postgaster*** (Fig. 11F, H): with rounded anterior sclerite (as) and pear-shaped median plate (mp). ***Endogyne*** (Fig. 11I): copulatory duct (cd) long, narrow, straight with tip reaching groove between posterior spiracles; apodemes (ap) short.

#### Etymology.

The specific name refers to the type locality and is a noun in apposition.

#### Distribution.

Known only from the type locality.

## Supplementary Material

XML Treatment for Camptoscaphiella

XML Treatment for Camptoscaphiella metok

XML Treatment for Camptoscaphiella shannan

XML Treatment for Camptoscaphiella trifoliata

XML Treatment for Camptoscaphiella zayu

## ﻿Additional information

### Author ORCIDs

Xiaohan Wang https://orcid.org/0009-0000-4614-9562

Yanfeng Tong https://orcid.org/0000-0002-4348-7029

Shuqiang Li https://orcid.org/0000-0002-3290-5416

### Data availability

All of the data that support the findings of this study are available in the main text.
